# Genetically Modified Microorganisms: Risks and Regulatory Considerations for Human and Environmental Health

**DOI:** 10.3390/microorganisms14020467

**Published:** 2026-02-14

**Authors:** Aaron Lerner, Arnon D. Lieber, Cass Nelson-Dooley, Andre Leu, Michelle Perro, Geoffrey Koch, Carina Benzvi, Jeffrey Smith

**Affiliations:** 1 Chaim Sheba Medical Center, The Zabludowicz Center for Autoimmune Diseases, Ahazmaout St. No. 8, Petach Tikva 4937908, Israel; 2 Independent Researcher, 47 Maale Uria St., Kfar Uria 9973500, Israel; 3 Health First Consulting, LLC, 1600 Indian Brook Way, Norcross, GA 30093, USA; info@healthfirstconsulting.com; 4 Regeneration International, 2543 Mossman Daintree Road, Lower Daintree, QLD 4873, Australia; 5 GMOScience, 769 Center Blvd., Suite 101, Fairfax, CA 94930, USA; 6 Petrichor Consulting Group LLC, Chico, CA 95965, USA; 7 Institute for Responsible Technology, P.O. Box 469, Fairfield, IA 52556, USA

**Keywords:** genetically modified microorganisms, environmental risks, human risk, gut microbiome, soil microbiome, infant microbiome, oral microbiome, microbial metabolites, international policy, regulatory considerations

## Abstract

Advances in affordable genetic engineering have accelerated the creation and large-scale environmental release of genetically modified microorganisms (GMMs). While beneficial applications exist, GMMs may present unique, long-term risks to human and environmental health. Unlike static chemicals, GMMs are biologically active, self-replicating entities capable of rapid mutation and global dispersal. Current regulatory frameworks place responsibility on each country to regulate GMMs, without a clear, coordinated international policy. This review details critical risk scenarios, including horizontal gene transfer to native species and the possible disruption of vital human microbiomes (gut, oral, and infant), which could increase resistance to degradation, promote traits that expand a microbe’s range of hosts or ecological niches, and enhance the production of novel metabolites with unexpected biological activity. In soil, GMMs may support the emergence of “super bugs” or destabilize carbon sequestration cycles, potentially impacting climate resilience. Engineered microbial enzymes in the food supply may also act as environmental drivers of autoimmunity. Given the limited understanding of microbial ecology, we propose a decision-based biosafety workflow emphasizing pre-release risk assessment and continuous post-release monitoring. We urge national and international regulators to adopt the precautionary principle to better protect human health and the environment from the potential negative outcomes of GMMs.

## 1. Introduction

New technologies may pose risks of harm to human, animal, and environmental health, and ideally should be vetted prior to release for commercial use or consumption. However, this is not always possible. The demands for a new technology may have to be balanced against policy, need, and cost. GMM as a “microorganism that has had its genetic material (DNA) changed intentionally” [1] is an outcome of such technology.

Currently, there is considerable disagreement about the proper regulations of GMMs. Pre-release safety testing, assessments for human and environmental impact, and monitoring frameworks are lacking. In the US, GMMs for commercial use are primarily regulated by the Environmental Protection Agency (EPA) as toxic substances, which is ill-suited for biological organisms. In addition, most other GMMs (not for commercial use) are unregulated and untracked. With the rapidly advancing landscape of genetic engineering technologies and broad access to these technologies across society, we must address the unique risks of genetically modified microbes and customize regulations based on current evidence and their impact on human and environmental health.

Soil microbes support healthy plants and food crops and are a key component of the Earth’s carbon cycle. Algae produce most of the planet’s oxygen. Fungal networks below the forest floor shuttle nutrients between trees and are the basis of a species communication system we are only beginning to understand [2]. Healthy, thriving microbial communities are essential for our planet’s future. Most microbial functions remain unknown; we have characterized less than 1% of the estimated one trillion varieties on our planet [3]. Yet wherever we look, we find that they play a vital role.

Microbes or microorganisms include bacteria, archaea, fungi, viruses, bacteriophages, and protists. A “microbiome” describes the collection of microbes living within a well-defined habitat or other environment [4]. Humans and their predecessors have been evolving with microbial inhabitants for 1.5 billion years [5]. The microbes that live in and on the human body are known as the “human microbiome” and function like an organ to support immunity, metabolism, detoxification, and resistance to infection. Lifeforms such as animals, plants, trees, fish, etc., have microbiomes. There can be distinct microbiomes for different parts of the body, such as the human respiratory microbiome, gut microbiome, or skin microbiome [6]. Microbiomes are found everywhere in the natural world.

### 1.1. Modern and Future Use of Genetically Modified Microorganisms

GMMs are being developed as consumable probiotics and targeted medicines. GMMs are used to make cheese [7], lactose-free milk, and B vitamins [8]. There are also efforts to create microorganisms to clean up environmental pollutants, including plastics in the ocean. GMMs are regularly produced in academic settings. With the widespread availability of CRISPR, even high-school science classes are rapidly becoming a source of GMMs.

GMMs are also being used in synthetic biology facilities (also known as synbio) to produce artificial genes, new gene arrangements, and more [9]. Synthetic biology is a field of advanced genetic engineering that seeks to “redesign life” by introducing unnatural (synthetic) chemical and biological capabilities into living organisms [10,11]. The organisms used in synbio fermentation to produce gene products are not intended for environmental release. Handling, disposal procedures, and human error, however, present a risk of escape.

The use of GMMs in agriculture, including biological control agents for plant disease [12] and bioremediation for soil, is expanding rapidly. The global market value in 2021 was $10.25 billion; it was predicted to nearly triple to $29.31 billion by 2029 [13]. Although most applications use naturally occurring microbes, the use of GMMs in agriculture is expected to increase dramatically.

Two prominent companies have gone public about their current use of GMMs on significant agricultural acreage. Pivot Bio acknowledges that it has released its GM bacteria on nearly 5 million acres, with as many as 5 trillion microbes per acre. Their product, Proven^®^, is the first genetically engineered microbe widely available in agriculture. It consists of live *Kosakonia sacchari* and *Klebsiella variicola* gene-edited microbes that continually fix nitrogen, instead of self-regulating their own nitrogen-fixing activity.

Bayer released a seed treatment called Poncho^®^VOTiVO^®^ 2.0 (now sold by BASF) that contains GM *Bacillus thuringiensis*. They announced in 2018 that it was spread across 10 million acres, with expectations that the number would quickly grow to 40 million [14,15], equaling the area where their Poncho^®^VOTiVO^®^ original version had been used.

There are numerous other applications of GMMs being proposed, researched, and developed. A 2023 research initiative from the Audacious Project, for example, aims to decrease methane emissions from livestock by genetically altering bacteria in the gut microbiome of cattle [16]. Scientists have genetically engineered a fast-growing marine bacterium to break down the plastic, polyethylene terephthalate, that comes from water bottles [17]. Industrial Microbes, Inc. develops genetically engineered microbes to consume methane gas and produce industrial chemicals [18]. Their noble goals notwithstanding, these and other applications may irreversibly release vast quantities of underregulated and understudied varieties of GMMs, which carry the potential for long-term unpredictable health and environmental damage.

This is not to say that GMMs are always a threat. Insulin has been biosynthesized through the use of recombinant DNA technology, inserting the human insulin gene into *Escherichia coli (E. coli* K-12*)*. Since the 1980s, this eliminated the reliance on animal sources (bovine and porcine pancreases) for insulin production and made insulin available to millions of people with diabetes [19]. Successful and well-executed applications of GMMs can save lives (human and animal), lower costs, and reduce environmental waste. However, affordable and accessible genetic engineering technologies are now widely available to both experts and novices alike. It requires a comprehensive approach to balance benefits and risks, and ensure safety, containment, and follow-up monitoring.

To illustrate the need for carefully devised laws and regulations, we provide examples of how GM bacteria might inadvertently cause harm. We review the risk to the human immune system and to the human gut, oral, and infant microbiomes, and the risks of GMMs to the soil microbiome and environmental health. This is a limited set of examples from which to extract principles that will apply to a broader range of microbes and environmental niches not discussed here, including fungi, algae, viruses, and marine ecosystems, among others. Further, we discuss the inherent technical risks associated with GMMs, international and national regulations, and an example of a successful release of a GMM, which can serve as a model.

The purpose of this article is to help responsible parties use science-based criteria to govern the creation, use, and environmental release of GMMs. We provide a checklist of key risk categories that can help inform responsible guidelines and requirements. Our hope is that this list will be further developed by stakeholders and scientists, and used as the basis for urgently needed safety-oriented regulations and protocols that consider the long-term impacts for human health and the natural world. Those making such decisions, including regulators, lawmakers, treaty delegates, companies, teachers, and school administrators, are often non-scientists. This article, therefore, includes explanations for laypersons alongside more formal scientific language and concepts.

As natural microbiomes have proven critical to human and environmental health, and GMMs carry a high level of unpredictability with the potential to produce long-term, significant ecological threats, we encourage regulators of GMMs to adopt the most conservative approaches at this time [20,21]. The domain of microorganisms is global; they freely travel across domestic and international boundaries [22]. Proper governance, therefore, may ultimately belong in the hands of international treaties.

### 1.2. Microbial Communities and Ecology

There are at least five unique characteristics of microbes that make the regulation of GMMs more difficult—and potentially more impactful—than genetically modified plants and animals [23].

1. Rapid replication. Unlike plants and animals that may require growing seasons and gestation periods to pass down traits to offspring, microbes under ideal conditions microbes can double their numbers in as little as 20 min [24].
2. Challenges with containment. Microbes are not easily contained. They can travel to distant and unexpected ecosystems and hosts and interact with a wide range of other microbes and organisms.
3. Gene transfer. Microbes might readily transfer their genes to other microbes (known as horizontal gene transfer), or receive genes transferred from GM or non-GM microbes. If they confer advantages, the transferred genes may continue to be passed on from mother to daughter cells, exponentially increasing their count.
4. Microbiomes are life-critical. Microbial communities are critical to the health and function of humans, animals, plants, and ecosystems around the planet.
5. Unknown complexities. Science has only identified perhaps one percent of the estimated one trillion microbes on the planet [3]. Furthermore, we have only begun to map the complex relationships within and between microbiomes, hosts, and ecosystems.

The unpredictability of the technology is made more problematic given that regulators cannot easily track the movements of these largely invisible organisms, whose impacts could be widespread, long-term, and difficult or impossible to remediate.

The widespread dissemination of bacterial resistance genes is a clear example of the extensive transfer of harmful genes among bacteria, some of which originate from foods consumed by humans [25]. Such concerns are not merely theoretical. In a rare and instructive example, Rosander et al. reported the deliberate removal of antibiotic resistance gene-–carrying plasmids from the widely used probiotic *Lactobacillus reuteri* ATCC 55730, underscoring that clinically relevant resistance determinants may persist in microorganisms intended for human consumption unless specifically identified and addressed [26]. This case demonstrates that mitigation is possible, but also highlights how such risks may remain undetected without systematic genomic and functional evaluation. Antibiotic resistance, however, represents only one aspect of potential concern. Lactic acid bacteria of aquatic origin proposed for probiotic use have been shown to exhibit additional traits with possible biological relevance, including gelatinase and hemolytic activities, as well as enzymes such as peptidases, phosphatases, and glycosidases [27]. These observations illustrate the inherent complexity of microbial phenotypes and reinforce the need for comprehensive, case-by-case assessment of microorganisms introduced into human or environmental microbiomes [26,27].

The human microbiome is normally stable and in harmony with the host unless disturbed by various stressors like medications, toxins, toxicants, infection, pH level, or significant changes in the diet [28]. GMMs could also be such a stressor, with particular concerns for human health. The human microbiome does not always go back to normal after antibiotic treatment or infection [29]. The loss of ancient beneficial human microbiota has been theorized to contribute to the increasing prevalence of modern-day chronic diseases [30]. Preservation of the microbiome may be pivotal for human health.

### 1.3. Rapid Advancement of Genetic Engineering Technologies Surpass Policy Updates

Recombinant DNA technology emerged nearly 50 years ago, in the years 1971–1973. Through the use of enzymes and various laboratory techniques, it became possible to combine DNA from two distinct species to create genes with new functions [31]. The resulting recombinant DNA is often propagated in a bacterial or yeast cell, which can easily replicate the engineered foreign DNA along with its own DNA [32].

Gene- editing techniques have broadened the set of microbes available for genetic engineering applications and the variety of people who can change DNA sequences. CRISPR-Cas9, in particular, is a simple, fast, versatile technology, making it possible to change the genome by removing, adding, or altering sections of the DNA sequence. It can turn genes on or off [33].

Numerous scientific and popular articles envision CRISPR’s revolutionary role in solving a long list of human and societal ills [34]. Increasing evidence, however, demonstrates that the process causes significant unpredictable changes in the genome, including additions, deletions, chromosomal shattering, and widespread mutations that do not occur naturally. *Nature* described the side effects observed in three CRISPR experiments on human embryos as “chromosomal mayhem” [35] referring to large DNA deletions and reshuffling of the genome.

The popularity and relative ease of CRISPR brought about a new wave of companies seeking to create solutions with GMMs. In addition, CRISPR reduced the cost and expertise required to create them [36]. Home hobbyists and high- school science students have joined the ranks of universities and businesses that now regularly produce novel genetically engineered microbes.

According to the Department of Homeland Security, “The speed of innovation has outstripped American regulatory policy and legislation; given the paradigm-altering potential of CRISPR and related technology, this disconnect must be closed.”

Rather than increasing regulatory requirements, numerous countries, including the US, Canada, UK, Japan, Australia, India, and others, have deregulated plant, animal, and/or microorganisms altered by gene- editing technology. The deregulated products are often limited to those that do not introduce foreign DNA from other species.

The lack of foreign DNA reduces some potential risks, such as transferring allergens. Gene- editing technologies, however, do introduce numerous unpredictable consequences for microbes, whether or not the process introduces foreign genes. They can:

- Create unintentional and undetected genetic aberrations; from point -mutations to large deletions and additions in the genome [37,38].
- Enhance the ability of microbes to survive, spread, adapt, replicate, and/or escape host defenses, and mutate [39].
- Introduce new substrates that microbes can use and alter their metabolic byproducts [40,41].
- Impact wild-type, non-GM microbes in a microbiome, as well as their survival or death (i.e., their prevalence) [42,43,44].
- Further, the technology can create countless combinations of GMMs, given the natural availability of thousands of types of microbes and the ease of defining CRISPR target sequences online.

## 2. Examples of Genetically Modified Microorganisms Risk Scenarios Contributed by Scientists and Physicians

### 2.1. GMMs Could Threaten Infant Gut Microbiome Acquisition, Immunological, and Neurological Development

How the microbiome is formulated in babies is directly related to the mother, the steward of the passing of microbial information directly to the fetus. The first three years of life are key in terms of the development of the host-microbe interactions, which directly impact the development of the baby’s immune system, gut health, and neurological development. This crucial period has long-lasting future effects on the health of the child [45].

Microbes are transferred from the mother to the fetus, and in infancy, via a vaginal birth, nursing, and skin-to-skin contact. Once believed to be sterile, studies show the placenta likely has a microbiome of its own, as well as the fetus. The microbes inhabiting the placenta are unique, divergent from vaginal microbiota, and interestingly, may resemble the oral microbiome [46]. Microbes have been detected in meconium (an infant’s first stool) and amniotic fluid, thus suggesting that the fetus may have its own microbiota [47,48]. It is now known that certain bacterial metabolites, as well as maternal antibodies, are key factors in the development of the fetal immune system [48,49].

The mother’s vaginal microbiome is transferred to the baby during vaginal birth, but not during Caesarean section birth (C-section) [50]. Babies born by C-section are more likely to suffer with asthma, allergies, eczema [51], inflammatory bowel disease, celiac disease, rheumatoid arthritis, diabetes [52], and metabolic syndrome [53] (a risk factor for diabetes and heart disease) later in life. This has led some researchers to believe that the mother’s vaginal microbiota is key in preventing these diseases. Babies born via C-section have more pathogenic microbes (which can harbor antimicrobial resistance) as compared to vaginally born babies [54]. Babies born by C-section have an increased risk of asthma, which may be mitigated by the gut microbiota [55].

Breastfeeding is another important stage when the mother’s microbiome is transferred to the infant to promote gut health and immune defenses. Breast milk is not sterile and can contain over 600 types of bacteria. Additionally, there are over 200 unique human milk oligosaccharides in breast milk, which are prebiotics or “food” for the microbes. Furthermore, a beneficial bacteria known as *Bifidobacterium infantis* encodes proteins that bind, transport, and digest different types of oligosaccharides. This produces short-chain fatty acids (SCFAs), beneficial bacterial byproducts, that are released into the gut. The acidic environment then creates a less hospitable environment for pathogens, thus protecting the infant. To turn the tables, the nursing infant via release of backwash can direct the mother’s breast to produce various immune substances that protect the baby from infection via the release of backwash [56,57,58,59].

It is noteworthy that upon comparing the infant microbiome on fecal smears from 100 years ago to the present, there has been a transition from a near monoculture of *Bifidobacterium infantis* to a microbial mix seen in non-breastfed infants, and/or older children. How GMMs could further affect create this alteration of the infant’s microbial landscape merits further study.

The gastrointestinal microbiome is critical for infant health [60]. It protects against pathogenic infections, promotes gastrointestinal development, and coincides with healthy neurological development. The infant microbiome helps build an informed and precise immune system—one that attacks infections, but does not attack harmless substances, as in the case of food allergies, or self-tissues, in the case of autoimmunity.

The importance of a healthy infant microbiome and how it is formed illustrate some of the complex biological systems and relationships involved—and that includes only’s only what we know about in this early stage of scientific discovery. It is essential that more information about the impacts of GMMs is obtained be understood before introducing them directly into this delicate system.

Limited knowledge exists on the risks of disruptions of not only to the infant—maternal microbial exchange, but to the future health of the baby. Genetically modified microbes could colonize the mother anywhere, from her oral cavity, to the placental microbiome, from the vaginal microbiome, to the areola, orto appearance in breast milk. Through by any of these routes, a GMM would have a direct line to the infant’s microbiome where it could colonize. Further, if altered genes from GMMs are incorporated via horizontal gene transfer into the microorganisms present in the mother or infant, it could affect the mother’s or infant’s health. A mother’s microbial make-up can lead to adverse pregnancy outcomes, threaten the livelihood of the fetus [61], and affect the infant’s oral health later in life [62].

There is currently no data evaluating how genetically engineered microorganisms could affect maternal breast milk or the effectiveness of breastfeeding. Many of these mechanisms are still poorly understood, such as retrograde flow from baby to mother during breastfeeding, also known as “baby spit backwash,” which can stimulate immune responses in the mother’s breast [56,57,58,59]. How GMMs can influence the placental, vaginal, or gastrointestinal microbiomes is unknown. Additionally, the effects of GMM on the infant’s own gastrointestinal development and immune regulation have not been evaluated.

All of these hypothetical risks must be thoroughly considered and investigated before releasing a GM microorganism with unknown or potentially harmful sequelae for mothers and their infants, especially given the broad spectrum of possible genetic modifications and their unique nature.

### 2.2. Genetically Modified Microorganisms Could Pose Threats to Human Oral and Systemic Health by Altering the Human Oral Microbiome

The oral microbiome is critical to human health, yet research is still in its infancy. Its location at the front entrance of the gastrointestinal tract makes it vulnerable to microbial, chemical, and allergenic exposures. Further, the oral cavity is believed to be an excellent environment for horizontal gene transfer between microorganisms [63]. The ramifications of unregulated, widespread use of genetic modification techniques could affect the oral microbiome and should be carefully weighed by scientists, healthcare professionals, and regulators.

The oral microbiome is the second-most biodiverse microbial population in the human body, housing at least 770 species [64]. Humans swallow approximately 140 billion bacteria into the gastrointestinal tract each day [65,66,67]. When balanced, the oral microbiome prevents disease, resists pathogenic infections, provides multi-layer immune defenses, and reduces inflammation. Friendly oral bacteria contribute up to 25% of a person’s total daily needs of the critical blood-pressure- lowering chemical, nitric oxide [68,69]. On the other hand, an imbalanced oral microbiome increases the risk of heart attack by nearly 50% [70]. It not only promotes cavities, gum disease, and heart disease [71], but an imbalanced oral microbiome has also been implicated in brain inflammation [72], lung infections [73], diabetes [74], head and neck cancers [75], preterm birth [74], and inflammatory joint disease [76,77].

New genetically modified microorganisms could pose the following risks to the oral microbiome: (1) destroying or displacing beneficial bacteria; (2) interferinge with the health-promoting functions of the oral microbiome; and/or (3) enhancing or reinforcinge pathogenic bacteria. For example, if genetically modified microbes were able to displace nitrate-reducing bacteria in the oral microbiome, it would promote high blood pressure and heart disease. If genetically modified microorganisms take up residence in the mouth, they could share genes with harmful oral pathogens via horizontal gene transfer, potentially creating more dangerous, more resilient, hardier, or treatment-resistant pathogens. Unfortunately, without proper regulation or study, these changes would be impossible to identify, track, or reverse.

### 2.3. Bioengineered Yeast Could Increase Risk of Human Gastrointestinal Infection with Pathogenic Clostridium Difficile

Gut microorganisms have influenced the resilience and survivability of mammals over evolutionary history [78]. Gut microbes have been shown to play an essential role in human health by influencing nutrient absorption [79,80,81], immune function, with transient and chronic inflammation [81,82,83,84,85], intestinal barrier integrity [86,87,88], metabolism [81,89,90,91], and mental health [92,93]. Over 1000 different species of bacteria from 30 different genera have been identified from the human gut [94,95], with cohabitation of many more species from other phyla. In a single individual, there are 108–1012 microorganisms per gram of fecal content [96]. Studies suggest that a more phylogenetically and metabolically diverse group of gastrointestinal microorganisms is associated with improved health while a community of bugs that is less diverse is seen in states of illness. Many are beneficial; that is, they produce digestive enzymes, bioactive acids (like lactic acid), and other protective compounds that keep the bowels, and the body functioning well. This incredibly complex interaction between human health and the gastrointestinal microbiome is the subject of numerous peer-reviewed publications [6,97,98,99,100].

Genetically engineered microorganisms could lead to imbalance and disease in the human gastrointestinal microbiome. Specifically, accidental release or spillage of the heavily bioengineered yeast, *Yarrowia lipolytica (Y. lipolytica)*, for example, could expose susceptible humans to harmful levels of succinate, thus triggering gastrointestinal infections with *Clostridium difficile (C. difficile)*, a highly contagious bacterium known to cause diarrhea and colitis.

*Yarrowia lipolytica* is a commensal yeast found as part of the microbiome of many animals, including the human gut and respiratory system. An aerobic organism, it thrives in oxygen-rich environments. Though normally harmless, it also can be an opportunistic pathogenic yeast in immune-compromised people [101].

*Y. lipolytica* can naturally metabolize a large variety of substrates such as glucose, fructose, and fatty substrates. *Y. lipolytica* is the second most commonly genetically engineered yeast, having had an instrumental role in the development of biomolecular engineering tools like CRISPR-Cas9- mediated gene editing [102], DNA assembler [103], and the Golden Gate Assembly system [104]. However, in the last decade, it was engineered to also utilize less common substrates [40] such as xylose, an ubiquitous monosaccharide (sugar) found in fruits, floral nectar, cereals, bread, potatoes, peas, carrots, and many fat-rich fermented food products such as cheese or salami. More recently, *Y. lipolytica* was engineered to use the common sugar xylose to manufacture large quantities of succinate [41].

Succinate is a normal intermediate of the Krebs (or tricarboxylic acid TCA) cycle. Succinate is also produced by anaerobic bacterial fermentation in the colon. In the healthy gut environment, succinate levels are low [105]. While it may seem natural and harmless, when succinate accumulates in extracellular tissue environments, it can promote disease states [105]. High succinate levels have been identified as a strong trigger for the overgrowth of the human pathogen *C. difficile* [106], a worldwide infectious disease challenge characterized by severe diarrhea.

Today, genetically engineered *Y. lipolytica* could colonize the human gut and overproduce succinate, setting the stage for *C. difficile* infection. Engineered traits such as resistance to acidic conditions could allow for new sections of the gastrointestinal tract (e.g., the small intestine) to be colonized by the microbe for the first time. In the presence of high xylose levels from the intake of common foods, genetically engineered *Y. lipolytica* could thrive on this novel preferred substrate in the gut. Certain vulnerable populations could be more susceptible, such as immunosuppressed patients or newborns, and prematurely born infants in particular. The infant gastrointestinal tract is oxygen-rich, an environment favored by *Y. lipolytica*. Succinate production in large quantities in unusual sections of the gut could promote inflammation and encourage *C. difficile* infections, as well as the population spread of this pathogen.

This is an example of how engineered microbial species, originally designed for industrial purposes, can function under new conditions (pH or temperature, for example) and produce unexpected consequences (chemical byproducts, cues, or interactions with other species) that are unique to those observed with natural species. Accidental release of *Y. lipolytica* could pose health and environmental risks. Proper containment facilities and monitoring are therefore needed [107].

### 2.4. Enzymes from Genetically Engineered Microorganisms Can Trigger Autoimmune Disease and Gastrointestinal Illness

Bioengineered bacterial enzymes pose risks to human health, including the immune system. The cross-linker enzyme, namely microbial transglutaminase (mTg), is a prototype example [108,109,110,111,112]. Transglutaminase enzymes are ubiquitous in nature: animals, plants, and microbes make them. Distinct from mTg, tissue transglutaminase (tTG) is a natural human enzyme involved in many essential cellular functions. However, this transglutaminase enzyme also has also been shown to play a role in a number of diseases, not limited to neurodegenerative, malignant, and autoimmune conditions, celiac disease included [113,114].

Microbial transglutaminase has many benefits for the food industry, especially when it comes to food texture and appearance. Unfortunately, mTg poses health threats because in the body, it acts like the human tTG enzyme, despite its divergent structure. mTg has the ability to change the structure and biochemistry of proteins in the human body through posttranslational modification, even though the enzyme is not naturally made by humans, but rather is made by microbes only. mTg is heavily used in the processed food industries for its role in deamidation and transamidation.

Gliadin is a class of proteins found in wheat. mTg, with its transamidated gliadin complexes, has been shown to be proinflammatory, immunogenic, allergenic, pathogenic and potentially toxic, hence, compromising public health [108,109,110,111,112,113,115,116,117,118,119,120]. When gliadin is deamidated and transamidated, it can trigger loss of immune tolerance and result in celiac disease in susceptible individuals [110]. mTg has been implicated in autoimmune disease [121], celiac disease [112,114,115,117,118,120], gastrointestinal dysbiosis [108,109], intestinal permeability [112], and even in neurodegenerative conditions [110,122]. The status of this enzyme as GRAS has been challenged, as well as warnings have been issued by several national regulatory authorities (Switzerland, Germany, Canada), yet it is widely available in processed foods around the world [108,110,111,112]. The estimated mTg doses to restructure 1 kg of food products is in the range of about 50–100 mg of the enzyme [109,110,112]. Altogether, a maximum daily consumption of mTg ranges up to 15 mg for any person who consumes processed foods. mTg sales worldwide in 2021 were $200 million in 2021 [121,123].

The food industry uses genetically modified microorganisms to produce mTg. Bioengineering increases the yield and efficacy of the enzyme production process, improves its thermostability, and enlarges the pH range the enzyme can withstand [124]. Currently, the engineered bacterium *S. mobaraensis* is the major food industry source, being the only mTg generally recognized as safe (GRAS). Since this source is insufficient for global-scale mTg production, genetic manipulation of the following species has also been explored: *Streptomyces lividans*, *E. coli*, *Bacillus subtilis*, and *Corynebacterium glutamicum*, as well as yeast species such as *Pichia pastoris* and *Yarrowia lipolytica*.

Some of mTg’s characteristics give it a broader reach in the human body and make it resistant to the usual physiological limits. mTg lacks regulatory elements for turning the enzyme on or off. Compared to the endogenous tTG, the exogenous mTg has fewer structural domains and has a much smaller molecular weight, which means it can operate through denser, cross-linked, gel-like substances and impaired tissues. It binds many substrates non-specifically and operates in a wider pH range than human tissue transglutaminase [111,119]. Bonds created by mTg are relatively resistant to usual breakdown mechanisms by protease enzymes. The enzyme operates at a higher reaction rate due to its improved cross-linking capacity, delivering a higher transamidation/deamidation ratio.

The mTg enzyme might alter the gastrointestinal mucosal layer [112] and/or the microbial biofilm, resulting in intestinal permeability [125], or compromising the microbial–-immune responses [126]. Transglutaminase enzymes usually catalyze mucin cross-linkages, thereby enhancing their protective antibacterial function [127]. However, the mTg-edited/or cross-linked mucosal proteins might decrease the protective functionality of the microbial biofilm or any other environmental gel [128]. Applying the genetically engineered microbial transglutaminase might affect the biofilm in the following ways: (1) alter the strength, (2) change the firmness and water-holding capacity, (3) decrease solubility, (4) increase surface hydrophobicity and reduced the bioactive molecules, and/or (5) suppress the CO2 and O2 permeability of the biofilm [128]. The mTg enzyme confers a serious potential risk to accelerate the development of autoimmune disorders. The most recent report of cross-reactivity and sequence similarity between mTg and various human epitopes strengthen the potential involvement of molecular mimicry in mTg- induced autoimmune and chronic brain conditions [122]. A leading mechanism of autoimmune disease, molecular mimicry is a case of mistaken identity whereby the immune system confuses self-proteins for harmful foreign proteins and sets off an attack against self-tissues.

Spreading, contamination, and leakage of those mTg-producing GMMs could result in devastation to humans and to the environment. This genetically engineered enzyme in the gut could impact many other prokaryotic and unicellular eukaryotes through horizontal gene transfer, including changing the human microbiome [23,129]. Cross-contamination of those GMMs represent a significant danger to the evolutionary environmental record as well as to animals, plants, and marine and terrestrial homeostasis.

Food products from genetically modified microbes pose risks to all living organisms when they are not thoroughly tested, studied, or regulated. The critics opposed to the current FDA GRAS and GMM policies are increasing in number [110,111,112,130,131,132,133,134,135]. Although numerous experts suggest that the GRAS status of mTg is no longer justified, engineered versions of mTg represent new potential threats to human health because they are more resistant to destruction by natural mechanisms (pH, temperature, protease degradation). The mTg global industrial usage is soaring;, the market size was valued at USD 136.51 million in 2024, and it is projected to grow from USD 148.65 million in 2025 to USD 266.99 million by 2033, growing at a CAGR of 9.8% during the forecast period (2025–2033) [136]. Food safety regulatory authorities should analyze the current knowledge and rigorously investigate foods produced with genetically modified microorganisms for large- scale human consumption in order to better safeguard consumer health and safety.

### 2.5. Genetically Modified Microorganisms Released in Soil Could Affect Climate Change and Disrupt Agricultural Systems

“The soil microbial community represents the greatest reservoir of biological diversity in the world” [137]. It is critical that policymakers, scientists, NGOs, and the wider public understand the fundamental importance of the soil microbiome in maintaining balance in agricultural systems, broader ecosystem health, and the fundamental process of soil formation [138,139]. Microbes play pivotal roles in soil formation by breaking down organic matter, weathering minerals, fixing nitrogen, contributing to soil structure, and participating in various soil processes. Their importance is reflected in their incredibly large numbers; there are an estimated 3 × 10^29^ bacteria living in soils worldwide. To put this into perspective, this equates to a few billion in a teaspoon of soil and the weight of about five cows in a hectare of soil [140]. Given the alarming rate of erosion of Earth’s topsoil [141,142], upon which all life depends, it is imperative to more fully characterize the soil microbiome and its roles in nutrient cycling, creating a critical soil structure, and climate change.

Understanding the soil microbiome’s immense metabolic capacity is key to harnessing it, not only for soil restoration but also for atmospheric carbon dioxide drawdown. With half of the global agricultural topsoils lost due to erosion—resulting from a combination of climate change and inappropriate agricultural practices—and a projected 90% at risk by 2050 (according to the UN Food and Agriculture Organization), humanity confronts an imminent crisis that could lead to the extinction of the human race. In this precarious scenario, microbes, and plants stand out as the most promising entities to assist humanity and sustain all life on Earth.

Nature has been degraded by human activities and restoring it will require a significant global human effort. However, introducing the unpredictable element of genetically modified microbes to enhance certain capacities, such as nitrogen fixation, into ecosystems already under considerable stress introduces exceptionally high risks. Nevertheless, GMO microbes have been, and are currently released at a large scales (millions of acres) into many parts of the world without rigorous risk assessments on how they might impact all the above processes, long-term or short-term.

In a comprehensive review conducted by Sudheer et al. [44], the advantages of genetically modified microbial inoculants are explored alongside their primary drawbacks. These drawbacks encompass challenges such as the limited survival of inoculants in the field, the potential transfer of harmful genes from GMM DNA to native soil bacteria, and environmental risks associated with genetically altered organisms. These risks include heightened pathogenicity and the emergence of pests or weeds [43]. The authors underscore a pivotal consideration when introducing microbial inoculants to the field: scientists must understand the impact on local soil microbial communities when foreign species (or genetically altered species) are introduced. Additionally, the review emphasizes the necessity for continuous monitoring of the behavior of GMMs. This monitoring process is not only costly but is also susceptible to stringent biosafety restrictions. Furthermore, many countries impose statutory exclusions to regulate the widespread release of genetically altered microbes into the field. This caution is rooted in the potential environmental risks, including the creation of new pathogens, harm to other soil microbes, and disruption of biotic communities and their associated processes.

Similarly, the role of invasive species and their detrimental effects on the soil ecosystem have received inadequate attention [143,144,145]. The introduction of biocontrol agents (beneficial microorganisms used to suppress plant diseases or pests) leads to changes in the functionality of the soil microbiome [143,144,145], and their long-term impact remains unclear. For instance, the introduction of commercial arbuscular mycorrhiza inoculants into the ecosystem may threaten the native soil fungal communities, potentially leading to profound impacts on ecosystem functioning. A salient example of how introducing a new soil organism into the environment can drastically alter it comes from earthworms. Earthworm species originating from Europe and Asia have been introduced to North American soils. Many celebrate these creatures as “nature’s soil engineers.” Earthworms are celebrated for their ability to decompose organic matter, mix and aerate soil, improve soil fertility, improve soil structure, etc. However, what is less known is that massive armies of these creatures are now completely transforming forest ecosystems by consuming organic matter and litter layers, homogenizing soil profiles, altering nutrient cycles, changing forest floor species composition and competing for food resources transforming the habitat of native species (including native earthworms) [146,147]. These exotic earthworms are seemingly harmless, but are altering the ecosystem by altering the selection pressures on plants and trees and the provisioning of other forest floor species. This illustrates that even the small changes that may seem unimportant can have very large effects decades later, and by the time problems are known, there is no way to effectively arrest these changes.

Microbes play essential roles in cycling nutrients [148] and contribute significantly to capturing not only carbon dioxide (CO_2_) but also other greenhouse gases in all ecosystems, especially methane and nitrous oxide [149,150]. Soil systems play a significant role in mitigating climate change by stabilizing and storing carbon, as soils contain the largest terrestrial pool of carbon, far exceeding that of standing biomass (vegetation) and atmospheric carbon [151,152]. Genetically modified microorganisms could become a threat in native ecosystems, disrupting the microbial processes that cycle and stabilize carbon, nitrogen, phosphorus, and other nutrients and greenhouse gasses in the terrestrial environment. They could cause disease or cause the extinction of vital microbes or plants in entire ecosystems. The stability and productivity of global soils are essential to the success of plants and, therefore, the success of all life on Earth. Disrupting soil ecosystems is a real threat that should not be ignored.

There is an even greater risk in unleashing genetically modified microbes into soil ecosystems because microbes can adapt and evolve continuously through processes such as horizontal gene transfer. Through this mechanism, a modified microbial gene from a GMM could be transferred to a native soil microbe, altering its genome and its ecological niche. In turn, a GM bacterium could incorporate a genetic element from a native soil microorganism, thereby expanding its ecological impact [42].

Governments around the world seek to reduce climate change (Intergovernmental Panel on Climate Change, IPCC) by limiting global warming to less than 1.5 °C (or 2.7 °F) by the year 2100. While one or two points on a thermometer may not seem like much to us, this small change will have profound ramifications worldwide, sparking drought, hunger, and conflict. If soil ecosystems become even more unstable due to disruptions of delicate cycling processes, the resulting changes could be catastrophic for humanity.

We have already observed unintended consequences for soil resulting from agricultural practices [153,154]. Altering the genome of one of the most common crop plants, wheat, through mutagenesis during the Green Revolution has resulted in vastly higher yields and a dramatic increase in the harvest index of this plant [155]. This has been beneficial for producing more food for an ever-growing population, but has resulted in far less straw residue being returned to soils to maintain carbon stocks. Similarly, the addition of mineral nitrogen fertilizers to agriculture has had a profoundly positive impact on the quantity of food produced in agriculture. However, these fertilizers also cause many problems, including increased nitrous oxide emissions from agricultural soils [156], environmental degradation [157,158], and faster oxidation of soil organic matter (through microbial decomposition) [159].

Microbes play essential roles in the environment—roles we are just now starting to understand. We are highly dependent on the many processes that take place, and it would be folly to intervene in these processes by altering the microbial DNA without rigorous control or regulation, given that there is still so much we do not know. Humanity has a poor track record for understanding the impacts of new technology, and this has never been truer than in the context of the soil microbiome.

The soil microbiome in agricultural systems is critical for capturing CO_2_. The IPCC has advocated for soil carbon sequestration as a carbon dioxide removal technology [160]. Microbes living on the roots of plants are able to take carbon dioxide from plant photosynthesis and convert it into soil organic carbon (SOC) [161,162,163,164,165]. Research has shown that scaling up soil carbon sequestration efforts can make a significant difference in mitigating and adapting to climate change [166,167]. Stimulating soil microbial communities can provide sustainable and less harmful solutions. For instance, regenerative agricultural practices that enhance the soil microbiota can increase the soil’s ability to sequester CO_2_ [164,168,169]. Regenerative grazing practices have been shown to sequester more CO_2_ in soil. [170,171,172]. The scaling up of best-practice regenerative agricultural systems could sequester 34.7 Gt of CO_2_ per year, enough to achieve negative carbon emissions [173].

The soil microbiome is critical to agriculture, horticulture, ecological equilibrium, and there is little doubt that a greater understanding and harnessing of microbial processes will be part of the answer to controlling climate change. However, there is far too much we do not know about the soil microbiome and the myriads of interconnected processes occurring in the soil, upon which all life depends. The massive current value of soil microbes in mitigating climate change (and tremendous future value) means that we must protect the biodiversity of the soil microbiome. We must strive to learn more and proceed with great caution. Releasing genetically modified microorganisms into the soil will have consequences, many of which we do not even understand, and could pose an existential risk to agriculture, global soil stability, and therefore to humanity.

### 2.6. Genetically Modified Microorganisms Could Encourage Soil “Super Bugs”

Although it is difficult to predict the impact of GMMs on soil, the introduction of non-native natural microbes provides some insight. Adding non-native or “foreign” microbes to soil can have a profound impact on the soil microbiome composition, diversity, and functioning, not predicted in advance. Furthermore, the longer the introduced microbes survive, the bigger the impact they have on the native soil microbiome [174,175]. Ultimately, most foreign microbes will die off when introduced to soil ecosystems that have significant diversity. The higher the diversity of the native community, the quicker these microbes die [176,177,178,179].

It is important to realize that despite their minute size, adding microbes constitutes a disturbance to the system [144]. Although the experiments performed above did not focus on GM microbes, it is highly probable that the introduction of GM microbes will impact the native soil microbiome, depending on the genetic modifications and the selection pressure exerted by the GMM on the native community. The questions that remain are to what extent these interactions will influence the health of the soil and for how long these changes will remain [143].

The introduction of GM crops with herbicide resistance has led to the natural development of herbicide-resistant “super weeds,” created by the constant use of these herbicides in the fields [180]. The GM plants are outfitted with a genetic trait that allows them to survive high amounts of herbicide application. Like the weeds above the soil, introducing GMMs into the soil biome may result in the emergence of superbugs. This depends on genetic selection, which is the evolutionary process by which certain traits become more common in a species than others. Depending on the type of GM microbes and the selection pressure put on the system to select for them, it is likely that the selection of super microbes will happen at a much faster rate than what we observed in super weed plants, which could lead to super soil microbes. Moreover, GMMs that harm soil biodiversity (by killing beneficial microbes) would be a significant threat because they would make the soil microbiome susceptible to invasion and disturbance [181]. The question we, as a society, should be addressing is how we can support and stimulate the natural, incredible diversity of beneficial soil microbes instead of modifying them or stamping them out [182,183,184,185].

## 3. Technical and Regulatory Considerations

### 3.1. Inherent Technical Challenges Working with Genetically Modified Microorganisms

There are inherent technical risks that arise when working with GMMs, as stated earlier. A central risk is effectively containing GMMs. Biocontainment strategies can reduce but not eliminate escape risk, as evolutionary pressure, mutational inactivation, microbial cross-interactions or human error may compromise safeguards during scale-up or long-term use [9,186,187,188]. Developers and manufacturers may need to destroy and/or dispose of large amounts of GMM waste without contaminating land or the water supply. Microbes act in communities and ecosystems, which extends their biological reach; yet, it is difficult to anticipate the off-target effects of a newly developed GMM. There are risks that horizontal gene transfer cannot be controlled, meaning that genetically modified DNA segments could be transferred to new species, providing a selective advantage over other microorganisms competing for the same niche [23,45,129]. Microbial DNA is not the only concern; microbes produce metabolites (including toxins), enzymes, and signaling molecules, which may have biological effects [40,41,108,109,110,111,112]. Testing the metabolic activity of GMMs adds further complexity to assessments.

**Figure 1:**
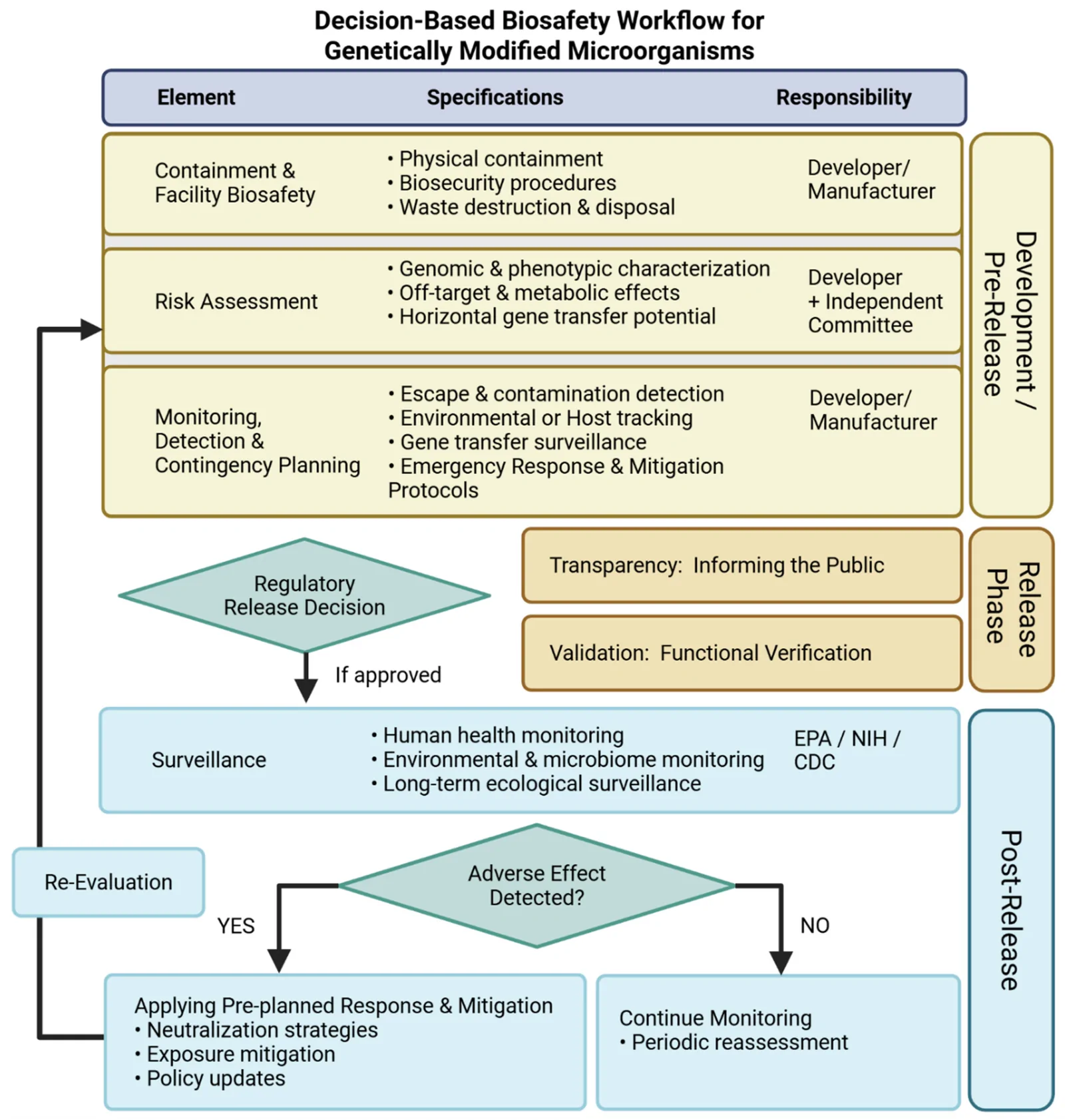
Decision-based biosafety workflow for genetically modified microorganisms. The figure illustrates a phase-based biosafety workflow for genetically modified microorganisms (GMMs) spanning pre-release, release, and post-release stages. The pre-release phase includes containment and facility biosafety, risk assessment, and monitoring with detection and contingency planning, addressing physical containment, biosecurity measures, waste management, genomic and phenotypic characterization, off-target and metabolic effects, and assessment of horizontal gene transfer potential. Functional validation and transparency measures are incorporated prior to a regulatory release decision. Following approval, post-release surveillance encompasses monitoring of human health, environmental and microbiome effects, and long-term ecological outcomes. Detection of adverse effects triggers the use of the contingency plan, allowing for rapid response and mitigation actions followed by policy updates and re-evaluation. Routine periodic reassessment ensures early detection of possible risk and effective mitigation procedures.

While there are numerous technical obstacles, these concerns can still be confronted as comprehensively as possible when developing a new GMM. To answer this need, we have included a comprehensive key risk categories checklist (Table 1) and a decision-based biosafety workflow (Figure 1). These tools are designed to illustrate the laws, regulations, and practices necessary to protect human health and the environment from the potential negative outcomes of GMMs. We also provide examples of alternative GMM risk mitigation technologies.

### 3.2. Contemporary Technologies for Genetically Modified Microorganisms Biosafety and Biocontainment

Concerns about the environmental release of GMMs have driven the evolution of biosafety strategies from reliance on physical containment (such as biosafety cabinets, controlled laboratory environments, and strict handling protocols) toward increasingly sophisticated intrinsic biocontainment approaches [193]. Intrinsic biocontainment refers to genetic safeguards engineered directly into the organism itself, often described as creating “suicidal microbes” that cannot survive outside defined conditions. Beyond physical containment methods, engineered genetic biocontainment tools include kill switches, toxin–antitoxin systems, orthogonal ribosomes, synthetic auxotrophy, CRISPR-based kill switches, genetic entanglement, and cell-free synthetic biology [194,195,196,197,198,199,200,201,202]. These approaches aim to restrict survival, reproduction, or genetic function outside controlled environments. Collectively, they demonstrate a shift toward multilayered biosafety architectures that complement physical containment. However, significant limitations remain in the reliability, scalability, and real-world deployment of these technologies. Kill switches and toxin–antitoxin systems are vulnerable to mutational escape, expressional leakage, and evolutionary trade-offs between containment strength and organism fitness, with escape frequencies increasing alongside circuit complexity [203]. Few of these sophisticated biocontainment technologies have been successfully deployed in real-world applications, with most engineered organisms still relying primarily on traditional physical containment or simple auxotrophies. This implementation gap reflects challenges in balancing robust containment with organism fitness, managing regulatory complexity, and scaling systems from controlled laboratory conditions to variable field environments.

### 3.3. Insufficient International Regulatory Framework for Genetically Modified Microorganisms

Regulatory systems for governing gene-edited organisms around the world have been reviewed elsewhere. The Cartagena Protocol on Biosafety to the Convention on Biological Diversity is a cornerstone international treaty governing the movements of living modified organisms from one country to another. However, each country is responsible for implementing and enforcing the Cartagena Protocol through national laws. Further, certain countries still have not entered into the treaty, including the United States. Other international groups offer guidelines or propose international standards for safe use of GMMs, such as the World Health Organization and the Organisation for Economic Co-operation and Development (OECD). The Codex Alimentarius Commission proposes international food safety standards for foods produced with GMMs, such as enzymes, vitamins, or other food ingredients, which influences the World Trade Organization. Information gathering, regulations, and enforcement depend on each country, without clear, overarching rules. This makes international regulations difficult to harmonize. To date, there has been no clear policy coordination effort on how to regulate genome-edited organisms in international organizations. We use the United States and the European Union as examples of how individual countries address GMM regulations.

### 3.4. Current State of Regulations in the United States

In the US, there is no federal legislation addressing GMMs as a category. No legislation requires GMMs to be treated with particular care or undergo a separate evaluation of potential environmental impact. In the US, responsibility for GMO regulation is divided between the Food and Drug Administration (FDA), the United States Department of Agriculture (USDA), and the Environmental Protection Agency (EPA). They rely on policies created prior to genetic engineering technology and are criticized as inadequate [204,205]. Most GMMs for commercial use fall under the purview of the EPA, which improperly regulates them as toxic controlled substances (industrial chemicals).

EPA reviews bacteria, fungi, algae, viruses, or protozoa that have been engineered to contain genetic material from organisms from a different genus. EPA believes these “intergeneric” microorganisms (novel microbes with DNA from two separate genera or taxonomic categories), have a sufficiently high likelihood of expressing new traits or new combinations of traits [206].

Under the Toxic Substances Control Act (TSCA), EPA only requires manufacturers of intergeneric GM microbes to submit a notice 90 days before commercialization of the product [207]. For non-intergeneric (gene-edited) GM microbes, only a premanufacturing notice [208] is required, which means they are treated with an even lower level of scrutiny. They do not properly address the risks associated with gene-edited GMMs, which do not contain foreign DNA. Field trials of GM microbes require only a TSCA Experimental Release Application submitted 60 days prior to the field test [207].

Regulating GMMs using methods designed for toxic chemicals is inappropriate and ignores the fact that these are biologically active organisms and critical to health and environment. Sixty to ninety days is not ample time for a careful regulatory analysis of human safety and environmental impacts. Because EPA only regulates GMMs created for commercial use, most other GMMs, including those produced by formal and informal research, students, and home hobbyists, are unregulated and untracked [209].

FDA regulates any GM microbes containing foreign genetic material that came from a pathogen. The US Department of Agriculture’s (USDA) Animal and Plant Health Inspection Services (APHIS) regulates GM viruses, bacteria, fungi, and parasitic plants that cause disease, injury, or damage to plants. USDA regulates these through either a permit or a notification process. A permit is required for field trials of GM crops that produce pharmaceutical or industrial chemicals, requiring either an Environmental Assessment or an Environmental Impact Statement.

The US government is not required to release information on GMM use, which makes third-party evaluation and monitoring difficult or impossible. Other than for specified pathogens, there are little to no regulatory restrictions on the type of microbe used, the nature of the genetic modification, the conditions for environmental release, or other important safeguards. Poor regulation, monitoring, and enforcement means that accidental releases may go undocumented.

### 3.5. Current State of Regulations in Europe

In the European Union, the approval of GM microorganisms is more rigorous and takes longer than in the United States. Before a product containing or produced using GMMs can be placed on the market and used in food or feed products, the European Food Safety Authority (EFSA) requires a scientific risk assessment [9,210]. This assessment includes two parts: 1) the characterization of the GMM and 2) the potential consequences modification may have on the safety of the product. The assessment analyzes the parental organism, the donor of the genetic material used, the genetic modification, and the final GMM and its traits. Additionally, the risk assessment evaluates the composition, nutritional value, potential toxicity/allergenicity, and impact of the product on the environment [9]. The final risk assessment then undergoes scientific evaluation to determine whether the GMM poses any safety issues. The conclusions of the scientific evaluation are then sent to European regulators who determine whether the product can be made available commercially [9]. The final determination of whether a GM microorganism can safely be released into the environment is based on the interpretation of regulators.

### 3.6. Successful Regulatory Case Study: Pseudomonas fluorescens HK44

#### Environmental Bioremediation and Biosensing Applications

While there are numerous gaps and pitfalls with GMM regulations, there are also cases of successful GMM release that can serve as models. *Pseudomonas fluorescens* strain HK44 is a landmark example of a GMM successfully regulated for environmental use, representing the first EPA-approved recombinant bacterium released for bioremediation [211]. The strain was engineered by inserting a bioluminescent gene code (luxCDABE from *Vibrio fischeri*) into a bacterial plasmid (a small, circular DNA molecule). It was capable of breaking down polycyclic aromatic hydrocarbon (PAH) environmental pollutants while emitting light as a real-time indicator of metabolic activity [212]. This dual function addressed a key limitation of conventional bioremediation—the reliance on costly chemical analyses such as gas chromatography–mass spectrometry to monitor pollutant degradation [211]. Beyond bioremediation, HK44 has served as a model organism for studying bacterial colonization, immobilization strategies, and bioluminescent kinetics under environmental conditions [213].

The regulatory approval of HK44 involved oversight by the U.S. Coordinated Framework for Regulation of Biotechnology. In 1995, a Pre-Manufacture Notification was submitted to the EPA under the Toxic Substances Control Act for an intergeneric microorganism, supported by extensive safety data including genomic characterization, antibiotic resistance profiles, pathogenicity testing, environmental persistence studies, and ecological risk assessments [214,215]. Parallel reviews were conducted by the USDA’s Animal and Plant Health Inspection Service under the Plant Pest Act and determined it was not a plant pest; the Department of Energy under the National Environmental Policy Act conducted environmental assessments as well [211,214]. EPA approval was granted in 1996 with monitoring conditions, followed by a controlled environmental release and long-term surveillance lasting up to 14 years—one of the longest documented tracking studies for any released GMM [211,216]. The results demonstrated effective PAH degradation, environmental containment, population decline without ecological harm, and no evidence of horizontal gene transfer to indigenous soil microbiota [211,216]. HK44 was approved only for contained research applications by EPA, not for commercial use; it has therefore never been marketed as a commercial bioremediation product, reflecting the stringent regulatory barriers that persist for environmental GMM applications even after successful proof-of-concept demonstrations. HK44 established a regulatory precedent for environmental GMMs and became a widely used research model, illustrating how flexible yet rigorous regulatory oversight can enable innovation while ensuring biosafety [213,215].

## 4. Conclusions

Microorganisms comprise the most complex and diverse systems in biology. Microbes and microbiomes coevolved with humans, animals, plants, and planetary ecosystems and are essential to their health and survival. Science is still in its infancy in understanding this foundational part of nature. Only about one percent of the earth’s microorganisms have been characterized [3], and even less is known about the impact of genes, gene regulatory networks, and metabolites within these kingdoms.

We provide GMM risk scenarios of soil organisms and human microbiomes in infants, the oral cavity, and the gut from experts in their fields. GMMs may impact the human microbiome by colonizing or interacting with the gut, mouth, or skin microbiome, or elsewhere [9]. The environmental release of GMMs raises concerns about sequelae for ecological systems [188].

We are not yet close to understanding the human microbiome and its health consequences. A total of 50% of the oral microbiome was unknown prior to the discovery of next-generation DNA sequencing technologies roughly 25 years ago [217]. Even with our limited understanding of this new field, evidence already links changes in the human microbiome with a significant number of human diseases [218,219]. It is therefore imprudent to risk contaminating, manipulating, or disrupting communities of indigenous microbes by manmade genetic codes that were not adequately tested. We should exercise extreme caution when considering the release of GMMs into water, soil, or the food supply.

The list of the most recent scientific publications that criticize the lack of adequate regulation and insufficient awareness of the public health risks of the GMMs is increasing rapidly [220,221,222,223,224,225,226,227]. The present narrative review summarizes the current various aspects of the GMM–environment–human interrelationship, in order to better protect human health and the environment from the potential negative outcomes of GMMs. It illustrates the need for new national and international laws and regulations governing the release of GM microbes. Policies must guard against uncontrolled results created from the random nature of experiments conducted in schools, unanticipated consequences from the commercial release of GM microbes, and the accidental escape of GM microbes used in the industry.

Besides its clear potential, the introduction of new genetic alteration and gene combinations created through genetic engineering has the potential to disrupt the functions, diversity, interactions, and impacts of microbes and microbiomes. This puts human and environmental health at risk. Worst-case scenarios include the promotion of diseases, risks to species survival, and damaged or collapsed ecosystems.

While quantitative risk assessment is complicated, the scientific community should nevertheless pursue efforts to quantify relationships between GMM release parameters (ranging from straightforward metrics such as concentration, duration, and application context to more complex measures such as multi-omics profiling approaches) and measurable risk indicators. Critically, because spatial mobility, evolutionary dynamics, and system-level uncertainties may remain fundamentally unquantifiable, effective governance requires continuous post-release monitoring, periodic reassessment, and robust mitigation preparedness in addition to quantitative pre-release evaluation.

Decisions and policies that oversee the creation, use, and release of GMMs must take these significant risks into consideration and err on the side of abundant caution. The regulation of GMMs becomes even more difficult due to their speed of replication, ability to mutate and travel, the ease with which genes are transferred between microorganisms, their small size and the difficulty in tracking them, and the potentially irreversible nature of environmental releases.

It is possible that a GMM originating from any number of countries could contribute to increased microbial pathogenicity or harm and spread rapidly throughout the world. Therefore, global treaties may be the appropriate method for establishing guidelines and laws.

Policymakers and regulators at every level, including those governing the use of GMMs in schools, labs, synthetic biology factories, and those intended for outdoor release, are urged to consult “Table 1. Key Risk Categories for Genetically Modified Microorganisms (Checklist).” Scientists and regulators must collaborate so that GMM policies reflect a comprehensive and up-to-date understanding of microbes, microbiomes, ecological networks, and all the ways that GMMs might cause unanticipated or irreversible harm to human health and the environment.

## Figures and Tables

**Table 1:** Key risk categories for genetically modified microorganisms (checklist).

Risk Category	Primary Concerns
Horizontal Gene Transfer (Outbound)	Engineered genes may transfer to native species, potentially enhancing pathogen virulence, altering commensal microbe characteristics, or creating novel environmental threats.
Horizontal Gene Transfer (Inbound)	Native genetic material entering GMMs may confer over-competitive traits, expand host range, or enable resistance to biological control mechanisms.
Novel Biological Signals and Interactions	GMMs may produce unexpected metabolites or signals that disrupt microbiome homeostasis, affect host immunity, trigger inflammatory responses, or enable colonization of unanticipated niches.
Altered Host or Microbial Metabolism	GMMs may interfere with native or pharmaceutical metabolism, particularly in the gut, requiring dose adjustments or alternative treatment modalities.
Virulence Enhancement	Mobile genetic elements (mRNA/sRNA systems) may increase pathogenicity of the GMM.
Containment Failure	Release of intermediate development versions may include environmental exposure to unexpected dosage, unintended mutations, antibiotic resistance genes, or uncharacterized genetic elements.
Unintended Genetic or Systems-Level Consequences	Unpredictable changes to genome, transcriptome, proteome, and metabolome may alter stress responses, membrane structure, environmental persistence, or regulatory pathways.
Diagnostic Complications	Modified organisms may require specialized detection methods, and consultation with reference laboratories during outbreak investigations.

Note: Based on references [23,186,187,189,190,191,192].

## Data Availability

No new data were created or analyzed in this study. Data sharing is not applicable to this article.
